# Pressure Pain Thresholds Across Nerve-Related and Muscular Sites in Frequent Episodic Tension-Type Headache: An Exploratory Case–Control Study

**DOI:** 10.3390/jcm15155843

**Published:** 2026-07-26

**Authors:** Rocío Carballo-Ponce, Leandro H. Caamaño-Barrios, Alberto Nava-Varas, Naiara Benítez-Aramburu, Ricardo Ortega-Santiago, Fernando Galán-del-Río, Juan Antonio Valera-Calero

**Affiliations:** 1 Escuela Internacional de Doctorado, Universidad Rey Juan Carlos, 28922 Alcorcón, Spain; r.carballo.2025@alumnos.urjc.es (R.C.-P.); albertonavavaras@gmail.com (A.N.-V.); 2 Escuela Universitaria Gimbernat Cantabria, Adscrita la Universidad de Cantabria, 39300 Torrelavega, Spain; leandro.caamano@eug.es (L.H.C.-B.); naiara.benitez@eug.es (N.B.-A.); 3 Department of Physical Therapy, Occupational Therapy, Rehabilitation and Physical Medicine, Universidad Rey Juan Carlos, 28922 Alcorcón, Spain; fernando.galandel@urjc.es; 4 Grupo de Investigación de Alto Rendimiento en Evaluación Multidimensional y Tratamiento del Dolor Crónico, Universidad Rey Juan Carlos, 28922 Alcorcón, Spain; 5 Department of Physiotherapy, Universidad Complutense de Madrid, 28040 Madrid, Spain; juavaler@ucm.es

**Keywords:** diagnostic accuracy, tension-type headache

## Abstract

**Background/Objectives**: Pressure pain thresholds (PPTs) are widely used to quantify hyperalgesia in tension-type headache (TTH). However, previous diagnostic accuracy research has mainly focused on muscular or segmental sites, while the discriminative performance of PPTs measured over peripheral nerves remains largely unexplored. This study primarily aimed to compare PPTs measured across nerve-related and muscular anatomical sites between women with frequent episodic TTH and headache-free controls. As a secondary exploratory objective, site-specific ROC analyses were performed to examine within-sample discrimination and derive sample-specific exploratory cut-offs. **Methods:** A cross-sectional exploratory study with a case–control sampling design evaluated 31 women with frequent episodic TTH (mean age: 19.6 ± 4.3 years) and 32 headache-free women (mean age: 22.1 ± 4.9 years). The groups differed significantly in age (*p* = 0.039). PPTs were recorded over peripheral nerves (greater occipital, median, ulnar, radial, tibial, common fibular), muscles (temporalis, tibialis anterior), 2nd–3rd interdigital hand space and the C5/C6 zygapophyseal joints. **Results:** Women with TTH showed lower PPTs than controls across all evaluated locations. However, after adjustment for age and Holm correction across the 20 anatomical sites, only the right greater occipital nerve remained statistically significant (*p* = 0.043). The right greater occipital nerve also yielded the highest area under the curve (AUC = 0.706; 95% CI 0.576–0.836) and was the only location whose AUC remained significantly different from 0.50 after correction for multiple testing (*p* = 0.040). Its sample-derived exploratory cut-off of 1.75 kg/cm^2^ showed low sensitivity (0.419; 95% CI 0.245–0.609) but high specificity (0.969; 95% CI 0.838–0.999). Although the corresponding LR+ was 13.419, its confidence interval was extremely wide (95% CI 1.866–96.525), indicating substantial imprecision. The LR− was 0.599 (95% CI 0.442–0.814). Overall, the operating characteristics varied considerably across locations and should be interpreted as exploratory. **Conclusions:** Women with frequent episodic TTH showed generally lower PPTs across nerve and muscle locations. However, after adjustment for age and multiple testing, the between-group difference was statistically supported only at the right greater occipital nerve. Although this location showed the highest AUC and high specificity at the sample-derived cut-off, its low sensitivity and the considerable imprecision of the LR+ estimate limit its interpretation. These findings reflect discrimination between selected participants with established TTH and headache-free controls, and should not be interpreted as evidence of clinical differential-diagnostic accuracy.

## 1. Introduction

Primary headache disorders are the most frequent form of headache [1]. Within primary headaches, migraine and tension-type headache (TTH) are the two dominant entities. Migraine is characterized by recurrent attacks of moderate to severe head pain often accompanied by transient neurological and systemic manifestations such as sensitivity to light and sound, cutaneous hypersensitivity, and digestive symptoms [2]. In contrast, TTH is a highly prevalent neurological disorder marked by bilateral, pressing or tightening pain of mild to moderate intensity, often described as a band or “helmet” around the head and usually lacking the prominent neurological features seen in migraine [3].

From an epidemiological standpoint, tension-type headache (TTH) is one of the most widespread neurological disorders worldwide. A large meta-analysis of population-based studies estimated that roughly one quarter of the global population live with an active TTH at any given time (pooled prevalence around 26%), with lifetime estimates exceeding 40% in some series and a 1-day prevalence of approximately 9%, meaning that hundreds of millions of people experience TTH on any given day. These studies also show important geographical variation, with active TTH prevalence ranging from about 11% in some Southeast Asia–East Asia–Oceania settings to over 30% in South Asia, and global summary estimates around 26% when all regions are combined [4]. In addition, it should be noted that episodic TTH is the most common presentation andan epidemiological study conducted in Denmark estimated prevalences of 46%, 39%, and 15% for infrequent episodic TTH (<14 headache days per year), frequent episodic TTH (15 to 179 headache days per year), and chronic TTH (>180 headache days per year), respectively [5].

Complementing these figures, Global Burden of Disease 2019 [6] data focused on individuals aged 15–39 years reported nearly 965 million TTH cases in 2019, representing an increase of about 37% in absolute numbers since 1990, with the highest case counts in middle-SDI regions and a positive correlation between TTH burden and sociodemographic development. Over the same period, TTH-related disability-adjusted life years (DALYs) rose from roughly 1.45 to 1.99 million (approximately 38% increase), while the age-standardized DALY rate showed only a small change (from 65.9 to 67.2 per 100,000), with disability peaking in the 35–39-year age group and modest sex differences most evident in the early thirties [7].

Pressure pain thresholds (PPTs) obtained by pressure algometry provide an objective estimate of mechanical pain sensitivity [8] and are widely used to quantify hyperalgesia in headache populations [9]. The importance of assessing PPTs is also supported by previous network-based analyses of patients with TTH [10]. The authors demonstrated that PPTs behave as tightly inter-related psychophysical markers. PPTs at cranio-cervical and distant sites cluster together and exhibit strong positive correlations with one another (highlighting the association between cervical spine and temporalis locations) while displaying also direct associations with gender (greater hyperalgesia in women), depressive symptoms and low vitality.

TTH remains a clinical diagnosis based on headache history and the diagnostic criteria established by the International Classification of Headache Disorders, third edition (ICHD-3) [3]. Therefore, pressure algometry is not proposed as a replacement for clinical diagnosis or as a stand-alone diagnostic aid. Instead, PPTs provide psychophysical information regarding mechanical pain sensitivity and may contribute to phenotyping the distribution of hyperalgesia and characterizing localized or widespread sensitization patterns in individuals with TTH [9,11,12,13]. Assessing PPTs across cranio-cervical, extra-trigeminal, and distal muscle and nerve locations may also provide exploratory mechanistic information regarding whether altered sensitivity is predominantly regional or extends beyond the symptomatic area [12,13].

Muscle and nerve PPTs may provide complementary rather than interchangeable biological information. Muscle-site PPTs primarily characterize mechanical pain sensitivity within the tested muscle and surrounding myofascial tissues. By contrast, PPTs measured over superficial nerve trunks are intended to assess peripheral neural mechanosensitivity, although pressure algometry cannot completely isolate neural tissue from the surrounding structures. In TTH, the greater occipital nerve is particularly relevant because upper cervical afferent input converges with trigeminal input within the trigeminocervical complex [12,14]. Conversely, reduced PPTs over extra-trigeminal and distal nerve trunks may indicate that altered nociceptive processing extends beyond the symptomatic cranio-cervical region [13,15,16].

On this basis, nerve-site PPTs were considered potentially more discriminative than previously investigated muscular or segmental sites for two reasons. First, the greater occipital nerve represents a structure directly related to trigeminocervical nociceptive processing. Second, extra-trigeminal and distal nerve sites may capture a widespread neural hypersensitivity pattern that cannot be explained solely by local pericranial muscle sensitivity. Previous studies had reported increased nerve-trunk mechanosensitivity in individuals with TTH, whereas diagnostic-accuracy studies had largely restricted PPT assessment to muscular or segmental sites [13,17,18]. Nevertheless, potentially superior diagnostic performance was not taken for granted and was tested empirically through site-specific ROC analyses.

Between-group differences in PPTs and the ability of PPTs to discriminate individual participants address different research questions. A lower group mean may indicate greater mechanical pain sensitivity at the population level but does not necessarily imply sufficient accuracy to classify individuals. Conversely, an AUC above 0.50 indicates some degree of separation between the groups included in a specific sample but does not, by itself, establish a clinically meaningful biomarker or diagnostic test. Furthermore, although reduced PPTs may be compatible with altered peripheral or central nociceptive processing, pressure algometry alone cannot determine the underlying pathophysiological mechanism or establish central sensitization [9,12].

Therefore, the primary objective of this study was to compare mechanical pain sensitivity across nerve-related and muscular sites in cranio-cervical, upper-limb, and distal lower-limb regions between women with frequent episodic TTH and headache-free controls. As a secondary exploratory objective, we examined the within-sample discriminative performance of each anatomical location and derived sample-specific exploratory cut-offs using the Youden index. Based on the aforementioned theoretical framework, we hypothesized that women with frequent episodic TTH would exhibit lower PPTs than headache-free controls across both nerve and muscle sites, reflecting a pattern consistent with regional and potentially widespread pressure hyperalgesia. We further hypothesized that some nerve-related anatomical sites, particularly the greater occipital nerve projection, might show numerically greater within-sample discrimination than muscular or segmental locations. However, these comparisons were considered exploratory, and no superiority between anatomical sites was prespecified or formally tested.

## 2. Materials and Methods

### 2.1. Study Design

A cross-sectional exploratory study with a case–control sampling design was conducted to compare nerve and muscle PPTs between women with neurologist-confirmed frequent episodic TTH and headache-free controls and to examine the within-sample discriminative performance of PPTs at different anatomical locations. The study was not designed to evaluate PPTs as diagnostic tests within a clinical differential-diagnosis population, because participants with other primary or secondary headache disorders and other causes of head or neck pain were not included as comparator groups. The diagnostic assessment, eligibility criteria, pressure-algometry procedures, and anatomical measurement locations were defined before data collection. However, the site-specific ROC analyses and derivation of exploratory cut-off values were conceived subsequently as a secondary post hoc analysis of the existing dataset and were not included in a prospectively registered diagnostic-accuracy protocol.

To ensure methodological rigor, the study followed the Updated List of Essential Items for Reporting Diagnostic Accuracy Studies (STARD) [19] and the Enhancing the QUAlity and Transparency Of health Research (EQUATOR) guidelines [20]. The assessment protocol was selected based on previously published pressure-algometry procedures and evidence showing altered mechanical sensitivity at cranio-cervical, extra-trigeminal, and distant anatomical sites in individuals with TTH [9,11,13]. Accordingly, the protocol was designed to examine three clinically and mechanistically relevant territories (the symptomatic trigeminocervical region, extra-trigeminal regions, and distant pain-free regions). Muscle and superficial peripheral nerve sites were included to compare potentially complementary manifestations of mechanical hypersensitivity. The complete protocol was not independently validated as a new measurement instrument; rather, its individual procedures were supported by previous evidence regarding the reliability of pressure algometry over muscular and peripheral nerve structures [21,22].

Although the study protocol was not prospectively registered, ethical considerations and protection of participants’ rights were reviewed and approved by a Local Ethics Committee (Cantabria Research and Drug Ethics Committee ID: 2016/104) prior to the initiation of data collection. The absence of prospective registration particularly affects the interpretation of the subsequent ROC analyses, which should be considered exploratory and hypothesis-generating.

### 2.2. Participants

Participants were consecutively recruited between February and December 2018 at a university-based center in Cantabria (Spain). All participants had a diagnosis of TTH, established by an experienced neurologist. The diagnosis was based on the third edition of the International Classification of Headache Disorders [3].

The diagnosis of frequent episodic TTH was established before pressure algometry by the same experienced neurologist in all participants. The diagnostic assessment included a clinical interview, review of headache history, evaluation of headache characteristics and associated symptoms, and physical examination, using the ICHD-3 criteria as the reference standard [5]. Consequently, the neurologist had no access to the PPT results at the time of diagnosis. Competing primary and secondary headache diagnoses were excluded using the ICHD-3 diagnostic framework and the findings of the neurological assessment. Imaging and other complementary investigations were not routinely included in the study protocol and were requested only when considered clinically indicated.

To be eligible, participants were required to have experienced at least 10 headache episodes occurring on 1–14 days per month on average for more than three months (≥12 and <180 days per year). Headache episodes had to last from 30 min to seven days and present at least two of the following characteristics: bilateral location, pressing or tightening non-pulsating quality, mild-to-moderate intensity (3 to 6 points on a 0–10 numerical pain rating scale [23]), and no aggravation by routine physical activity. Participants were also required to report no nausea or vomiting and no more than one of photophobia or phonophobia. The headache could not be better accounted for by another ICHD-3 diagnosis. Participants completed a prospective four-week headache diary to complement the neurologist-established diagnosis and to record the number of headache days, headache intensity, and duration of each attack [13]. The requirement that the frequency pattern had been present for more than three months was established from the clinical history obtained during the diagnostic interview. To minimize the potential acute influence of headache symptoms and analgesic use on PPT measurements, participants with frequent episodic TTH were assessed on a headache-free day and were instructed to avoid analgesic medication or other analgesic measures during the 24 h preceding the assessment.

A control group without a history of headache or headache episodes during the previous year was recruited. Exclusion criteria for the control group were the same as described above. All participants read and signed the informed consent form prior to inclusion.

### 2.3. Sample Size Calculation

Sample size calculation was determined using the G*Power software (v.3.1. for Mac OS) based on detecting a moderate-to-large effect size (0.75) between the tension-type headache and healthy control groups, using a two-tailed test, an alpha level of 0.05, and a statistical power (1−β) of 90%. This resulted in a required minimum of 30 participants per group.

### 2.4. Pressure Pain Sensitivity

An electronic pressure algometer (Somedic AB, Farsta, Sweden) fitted with a circular probe of 1 cm^2^ was used to assess PPTs. The device was calibrated in accordance with the manufacturer’s recommendations. Pressure was applied at approximately 0.3 kg/cm^2^ per second. Participants were instructed to press a handheld switch as soon as the pressure sensation first became painful, at which point the application was immediately stopped.

All measurements were performed by the same physiotherapist, who had more than 10 years of clinical and research experience in pressure algometry. Before data collection, participants completed a familiarization procedure to ensure that they understood the distinction between pressure and the first painful sensation.

Ten anatomical structures were assessed bilaterally (to characterize mechanical pain sensitivity on both sides but no dominant, more symptomatic, or clinically relevant side was prespecified, and laterality was not included as a mechanistic hypothesis), resulting in 20 side-specific measurement locations and 60 pressure applications per participant. The evaluated structures comprised the temporalis and tibialis anterior muscles; the C5/C6 zygapophyseal joints; the second–third interdigital hand space; and the greater occipital, median, ulnar, radial, tibial, and common fibular nerves. The measurement sites were grouped according to anatomical region. The cranio-cervical region included the temporalis muscle, greater occipital nerve, and C5/C6 zygapophyseal joint. The upper-limb region included the median, ulnar, and radial nerves and the second–third interdigital hand space. The distal lower-limb region included the tibialis anterior muscle and the tibial and common fibular nerves. The greater occipital nerve was considered a cervical peripheral nerve and not a trigeminal structure.

Participants were positioned according to the anatomical structure being assessed so that the tested tissues remained relaxed and accessible following the instructions from previous studies describing the procedures for these locations [22,24,25,26,27]. Each anatomical landmark was identified by palpation and marked with a skin pencil before testing. The algometer probe was positioned perpendicular to the skin surface over the predefined measurement point.

For the cranio-cervical measurements, the participant’s head was supported in a neutral and relaxed position. The temporalis muscle was identified by palpation during a brief dental clench, after which the participant was instructed to relax the jaw and avoid tooth contact. The measurement point was located over the central portion of the temporalis muscle belly. The greater occipital nerve was identified by palpation along a horizontal line passing through the external occipital protuberance. Its location varied according to individual anatomy but was situated, on average, approximately 4 cm lateral to the external occipital protuberance, with a previously reported range of 1.5–7.5 cm. For the C5/C6 zygapophyseal joint, the participant was positioned prone with the head supported in a neutral position. The C5/C6 articular pillar was identified by palpating the lower cervical spinous processes and moving laterally over the corresponding zygapophyseal joint.

For the upper-limb measurements, the participant was positioned supine or comfortably seated, with the arm fully supported and the shoulder, elbow, forearm, and hand relaxed. The median nerve was assessed in the cubital fossa, medial and immediately adjacent to the biceps tendon. The ulnar nerve was assessed within the groove between the medial epicondyle and the olecranon. The radial nerve was assessed where it passes through the lateral intermuscular septum, between the medial and lateral heads of the triceps, at the middle-to-distal third of the arm. For the second–third interdigital hand site, the hand was supported in pronation and the probe was applied over the predefined dorsal point between the second and third metacarpal regions.

For the distal lower-limb measurements, the limb was supported so that the knee, ankle, and surrounding musculature remained relaxed. The tibialis anterior muscle was assessed with the participant supine, over the upper third of the muscle belly, approximately 5 cm distal to the tibial tuberosity and lateral to the anterior tibial border. The tibial nerve was assessed with the participant prone and the knee comfortably supported in slight flexion, at the point where the nerve courses through the popliteal fossa, lateral to the popliteal artery. The common fibular nerve was assessed posterior to the fibular head, where the nerve passes around the fibular neck before continuing anteriorly.

The anatomical structures were evaluated following the same standardized cranial-to-caudal sequence in all participants. Although the order of anatomical sites was systematic (not randomized), the side assessed first was randomized for each participant (after which the corresponding contralateral site was evaluated). Bilateral measurements were obtained to characterize mechanical pain sensitivity on both sides; no dominant, more symptomatic, or clinically relevant side was prespecified, and laterality was not included as a mechanistic hypothesis.

Three trials were performed at each side-specific location, with a 30 s interval between measurements, and the mean of the three trials was used for the statistical analysis. The repeated measurements were collected to obtain a more stable estimate of PPT [21]. The physiotherapist was not informed of the participants’ formal group allocation and had no access to the neurological assessment, clinical records, headache diaries, or diagnostic results before completing the algometry procedure. Nevertheless, complete assessor masking could not be guaranteed because participants could inadvertently disclose headache-related information during interaction. The success of masking was not formally assessed.

Participants with frequent episodic TTH were assessed on a headache-free day and were instructed to avoid analgesic medication or other analgesic measures during the 24 h preceding the assessment.

### 2.5. Statistical Analysis

Statistical analyses were carried out using the Statistical Package for the Social Sciences (SPSS) v.29 for Mac OS (IBM Corp., Armonk, NY, USA). A two-sided *p* value < 0.05 was considered statistically significant for all tests.

Descriptive statistics were calculated separately for the TTH and headache-free control groups at each anatomical location. Right- and left-side measurements were analysed separately because they were acquired as distinct observations at each anatomical location. Nevertheless, side-specific analyses were considered exploratory, and no inference regarding a lateralized mechanism was prespecified. Because the groups differed significantly in age, between-group differences in PPTs were examined using analysis of covariance, with group as the fixed factor and age as the covariate. Age-adjusted estimated marginal means were calculated at the sample mean age of 20.90 years. Adjusted mean differences were expressed as controls minus TTH and reported with their 95% confidence intervals. Partial eta squared was reported as the effect-size estimate. Given that 20 anatomical locations were evaluated, the Holm procedure was applied to the 20 age-adjusted between-group comparisons to control the family-wise type I error associated with multiple testing [28].

The within-sample ability of PPTs at each anatomical location to discriminate between the two selected study groups was explored by constructing receiver operating characteristic (ROC) curves and calculating the AUC. No prespecified sensitivity or specificity thresholds were used to define clinical acceptability because the study did not evaluate a predefined rule-in or rule-out application of PPT testing. Sensitivity, specificity, and likelihood ratios were reported descriptively for the sample-derived cut-offs and interpreted together with the AUCs and their 95% confidence intervals [29]. The Holm procedure was also applied separately to the 20 hypothesis tests evaluating whether each AUC differed from 0.50. No formal pairwise comparisons between the AUCs of different anatomical locations were performed. Therefore, differences in the observed AUC values across sites were interpreted descriptively and were not considered evidence that one location performed significantly better than another. Statistical significance against an AUC of 0.50 was interpreted as evidence of within-sample separation from chance, but not as evidence of clinically meaningful or acceptable discrimination. The optimal cut-off point for each PPT site was determined using the Youden index, and the corresponding sensitivity, specificity, and positive and negative likelihood ratios (LR+ and LR−) were reported. The cut-off values were derived and evaluated within the same sample, and no separate internal or external validation cohort was available. Therefore, these thresholds were considered sample-derived exploratory cut-offs and not clinically validated diagnostic thresholds.

## 3. Results

**Figure 1:**
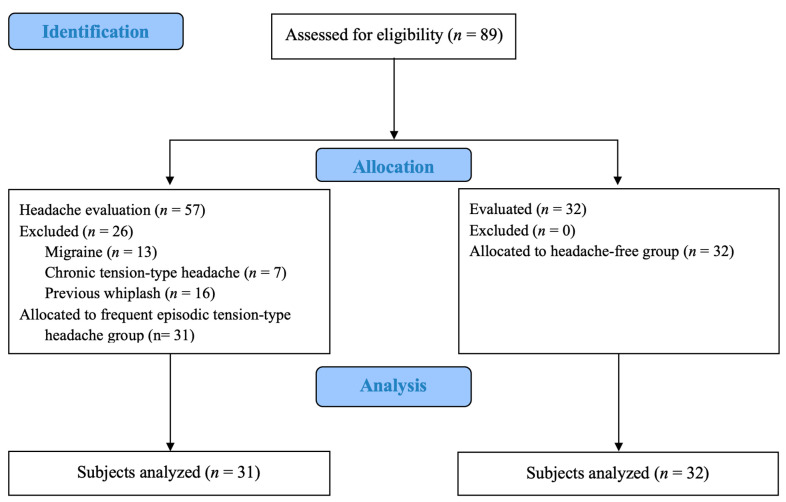
Participants’ flow chart.

A total of 63 female participants were included in the analysis. The identification, allocation and analysis process is illustrated in Figure 1. Although recruitment was not restricted by sex and sex was not an eligibility criterion, only women volunteered, met the eligibility criteria, and were ultimately enrolled. The final sample comprised 31 women with frequent episodic TTH and 32 headache-free controls.

Age differences were observed between the groups (control group 22.1 ± 4.9 years; TTH group 19.6 ± 4.3 years; *p* = 0.039). A description of the clinical characteristics of the sample with TTH is displayed in Table 1.

Across all evaluated locations, the TTH group showed descriptively lower PPT values than the headache-free control group. The overall multivariate effect of group across the 20 PPT outcomes was not statistically significant after adjustment for age (Pillai’s trace = 0.413; F = 1.442; *p* = 0.158; partial η^2^ = 0.413). In the site-specific analyses, the largest age-adjusted difference was observed at the left tibialis anterior muscle (adjusted mean difference = 1.50 kg/cm^2^; 95% CI 0.43–2.57), while the largest effect size was observed at the right greater occipital nerve (F = 10.270; partial η^2^ = 0.146). After applying the Holm procedure across the 20 anatomical locations, only the right greater occipital nerve remained statistically significant (adjusted mean difference = 0.74 kg/cm^2^; 95% CI 0.28–1.20; *p* = 0.043). The right posterior tibial nerve and right tibialis anterior muscle showed the next largest effect sizes, although neither remained statistically significant after correction (*p* = 0.056 and *p* = 0.057, respectively). Age-adjusted differences, effect-size estimates, and Holm-adjusted *p* values are presented in Table 2. Because no symptomatic or dominant side was prespecified, the better performance observed on the right side should not be interpreted as evidence of a true lateralized mechanism. The side-specific finding may reflect sampling variability within the relatively small study sample.

The ability of PPTs to differentiate participants with TTH from headache-free controls was limited overall. The right greater occipital nerve showed the highest observed AUC in this sample (AUC = 0.706; 95% CI 0.576–0.836) and yielded a sample-derived exploratory cut-off of 1.75 kg/cm^2^, with a sensitivity of 0.419 (95% CI 0.245–0.609) and a specificity of 0.969 (95% CI 0.838–0.999). The corresponding LR+ was 13.419, but its confidence interval was extremely wide (95% CI 1.866–96.525), indicating considerable statistical instability. The LR− was 0.599 (95% CI 0.442–0.814). After Holm correction across the 20 AUC hypothesis tests, this was the only location whose AUC remained significantly different from 0.50 (*p* = 0.040). The left temporalis muscle and left C5/C6 zygapophyseal joint showed AUCs of 0.640 (95% CI 0.502–0.778) and 0.638 (95% CI 0.501–0.774), respectively. Although their unadjusted confidence intervals narrowly excluded 0.50, neither remained statistically significant after Holm correction (*p* = 0.752 for both), and these findings should not be interpreted as evidence of clinically meaningful discrimination. Bilateral tibialis anterior muscles and the right tibial nerve showed AUCs ranging from 0.654 to 0.662, but these findings did not remain statistically significant after correction for multiple testing. Because no formal pairwise comparisons between AUCs were conducted, the numerical differences among anatomical locations should be interpreted descriptively. Cut-off values varied across locations, and their operating characteristics showed considerable uncertainty. Likelihood ratios were retained to provide transparent and complete reporting of the apparent performance observed in the derivation sample rather than as evidence of clinical diagnostic utility. Site-specific AUCs, 95% confidence intervals, exploratory cut-off values, and discriminative indices are presented in Table 3.

**Figure 2:**
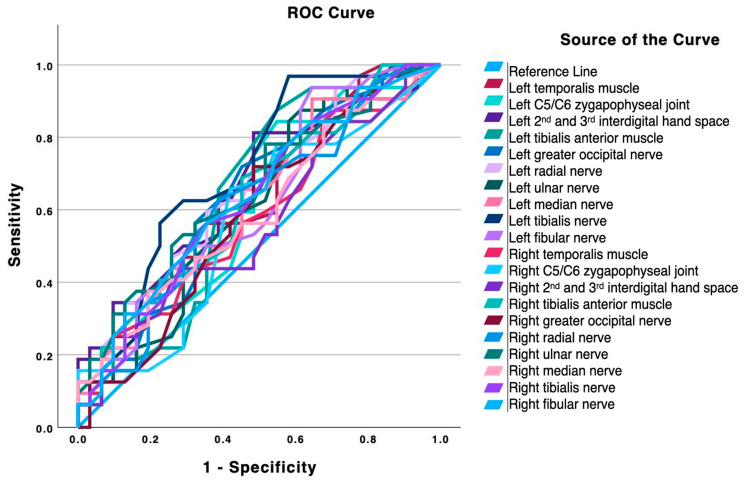
Receiver operating characteristic (ROC) curves for pressure pain thresholds measured over different anatomical sites to discriminate women with frequent episodic tension-type headache from headache-free controls. Each coloured line represents one anatomical location; the diagonal line indicates no discriminative capacity.

ROC curves (Figure 2) showed that most curves remained close to the diagonal reference line, indicating limited within-sample separation for most anatomical locations. The right greater occipital nerve had the highest observed AUC, while the tibialis anterior muscles and right tibial nerve showed some of the next highest numerical AUC values. However, no formal statistical comparisons between the AUCs were conducted, and the curves should therefore be interpreted descriptively rather than as evidence that any site performed significantly better than another.

## 4. Discussion

In patients with TTH, multiple reports have shown consistently lower PPTs over cranio-cervical structures than in headache-free controls, especially at the upper trapezius (midpoint between C7 and the acromion) and suboccipital musculature [9,13,26,30]. These reductions in PPT may be consistent with heightened mechanical pain sensitivity and altered nociceptive processing. However, PPT measurements alone cannot determine whether the observed differences originate from peripheral sensitization, central sensitization, altered descending pain modulation, or a combination of these mechanisms [9,12]. Importantly, similar patterns of decreased PPT can also be observed at extra-cephalic sites in some studies, suggesting that in a subgroup of patients the hyperalgesia is not purely regional but may represent more generalized changes in pain modulation [31,32,33]. Despite this, most investigations report only weak or inconsistent associations between PPT values and clinical variables such as headache frequency, intensity, or duration, supporting the view that PPT captures a relatively stable characteristic of nociceptive processing rather than a simple proxy of symptom load [34]. The systematic review by Castien et al. [9] also underlines the considerable heterogeneity in the selection and anatomical definition of measurement points and calls for standardized protocols focused on well-defined sites to improve comparability and to better determine which regions are most informative for detecting sensitization in headache disorders.

In this context, women with frequent episodic TTH showed descriptively lower PPT values than headache-free controls across all evaluated nerve and muscle locations. However, the overall multivariate group effect was not statistically significant after adjustment for age. In the site-specific analyses, only the right greater occipital nerve remained statistically significant after Holm correction (*p* = 0.043). The largest additional effect sizes were observed at the right tibial nerve and right tibialis anterior muscle, although these findings did not remain significant after Holm correction. Therefore, the results support a consistent descriptive pattern of mechanical hypersensitivity but provide more limited inferential evidence for site-specific between-group differences than the uncorrected analyses suggested. Regarding diagnostic performance, the right greater occipital nerve showed the highest AUC and was the only location whose AUC remained significantly different from 0.50 after correction for multiple testing.

Although the right greater occipital nerve remained statistically significant after correction for multiple testing, statistical significance should not be equated with clinical diagnostic usefulness. Similarly, the unadjusted confidence intervals for the left temporalis muscle and left C5/C6 zygapophyseal joint narrowly excluded 0.50, but neither AUC remained statistically significant after Holm correction. These findings illustrate that nominal separation from chance and clinically meaningful classification performance are distinct concepts. Its AUC was 0.706 (95% CI 0.576–0.836), indicating only modest separation between the two selected study groups and considerable overlap at the individual level. At the sample-derived cut-off of 1.75 kg/cm^2^, the right greater occipital nerve showed high specificity (0.969) but low sensitivity (0.419), indicating that a substantial proportion of participants with TTH would not be identified by this threshold. Although its LR+ was 13.419, the extremely wide 95% confidence interval (1.866–96.525) indicates substantial imprecision and statistical instability. Its LR− of 0.599 (95% CI 0.442–0.814) does not support a clinically useful rule-out function. Therefore, the apparently favourable specificity and LR+ should not be interpreted as evidence of a reliable rule-in test. Accordingly, these PPT measurements should not be considered diagnostic tests for TTH. Rather, they may provide exploratory and complementary information regarding the distribution of mechanical pain sensitivity. These operating characteristics represent apparent performance within the derivation sample and may be less favourable in new participants. The site-specific cut-off values should also be interpreted cautiously because they were derived and evaluated in the same sample and have not been independently validated.

These findings should be interpreted at four distinct levels. First, it should be noted that headache-related outcomes may also be influenced by broader demographic, behavioural, and environmental contexts. A recent 14-year time-series analysis of 5127 primary care consultations examined the associations of meteorological variables with cluster headache, TTH, and unspecified headache consultations using ETSX and ARIMAX models [35]. Climatic factors showed limited and headache subtype-specific associations: only average temperature and wind direction were independently associated with TTH consultations, whereas demographic variables, particularly sex, showed more consistent associations with healthcare utilization. These findings reinforce the multifactorial nature of headache disorders and demonstrate the value of long-term objective healthcare databases for examining environmental influences. However, consultation frequency reflects healthcare-seeking behaviour rather than individual headache occurrence or experimental pain sensitivity. Therefore, these results cannot be extrapolated directly to the PPT differences observed in the present study, but they highlight the need to consider demographic, environmental, and contextual factors when interpreting headache-related outcomes. Second, the descriptively lower PPT values indicate a group-level pattern of greater mechanical pain sensitivity but do not demonstrate that all anatomical locations were affected. Third, possible trigeminocervical or widespread sensitization represents a pathophysiological interpretation rather than a direct conclusion of the PPT measurements. Lastly, the ROC analyses quantify the degree of separation between the two selected study groups but do not establish that PPTs can accurately classify individual patients in clinical practice. Although the right greater occipital nerve showed the highest AUC, a value of 0.706 represents only limited discrimination and should not be considered evidence of a clinically meaningful diagnostic biomarker.

### 4.1. Nerve Tissues Mechanosensitivity at Nerve-Related Anatomical Sites

The hypothesis that PPTs measured at nerve-related anatomical sites might show greater discrimination than muscular or segmental sites was not confirmed. The right greater occipital nerve projection yielded the highest observed AUC in this sample; however, no formal pairwise comparisons between AUCs were conducted, and this finding does not demonstrate statistically superior performance. Furthermore, laterality was not prespecified, and participants were not classified according to a dominant or more symptomatic side. Given the generally bilateral nature of TTH, the isolated right-sided result may reflect random sampling variation rather than a lateralized trigeminocervical mechanism.

PPT measurements obtained over superficial nerve courses should also be interpreted as regional pressure-sensitivity measures rather than selective assessments of nerve tissue. Pressure applied at these sites stimulates not only the underlying nerve but also skin, subcutaneous tissue, fascia, muscle, vascular structures, and other adjacent tissues. Therefore, the lower PPT observed at the right greater occipital nerve projection cannot be attributed specifically to altered neural tissue sensitivity. It indicates greater regional mechanical pain sensitivity at this anatomical location within the selected sample. Although trigeminocervical convergence provides a plausible anatomical framework, the finding does not establish central sensitization, selective nerve dysfunction, or a specific pathophysiological mechanism.

Previous studies have similarly reported lower PPTs at anatomical sites overlying the supraorbital and other peripheral nerves in adults and children with TTH [15,16]. These findings support the presence of altered regional pressure sensitivity at nerve-related sites but do not demonstrate that the nerve tissue itself is selectively affected. Experimental evidence that greater occipital nerve blockade modulates nociceptive signalling within the trigeminocervical complex provides anatomical support for convergence between upper cervical and trigeminal afferents [14]. However, the therapeutic or neurophysiological effects of a nerve block do not validate PPT assessment at the same location as a diagnostic marker.

Regarding upper-limb and distal lower-limb nerve-related locations, women with TTH showed descriptively lower PPTs at several sites. However, none of these between-group differences remained statistically significant after Holm correction. Taken together, these observations are compatible with a possible pattern of widespread pressure hyperalgesia, but they do not establish generalized hypersensitivity or central sensitization. Alternative explanations include regional differences in the tissues compressed by the algometer, measurement variability, psychological or contextual influences, and residual confounding from factors that were not recorded. These findings should therefore be considered exploratory and require confirmation in larger independent samples. A study conducted by Fernández-Mayoralas et al. [16] found increased sensitivity in all trigeminal and extra trigeminal nerves assessed in children with episodic TTH. Our findings contrast with those reported by Romero-Godoy et al. [36] who did not find extra trigeminal changes (i.e., radial nerve) in subjects with chronic TTH using PPTs or their interaction with other sensitivity-related variables. These differences may be partly attributed to variations in headache subtype and the measurement instruments used.

Concerning the ROC analyses, the right greater occipital nerve showed the highest, although still limited, discriminative performance and was the only nerve location whose AUC remained significantly different from 0.50 after correction for multiple testing. Its sample-derived cut-off of 1.75 kg/cm^2^ showed high specificity but low sensitivity. Although the LR+ was comparatively large, its extremely wide confidence interval indicated substantial uncertainty and prevented a clinically meaningful interpretation. The LR− did not indicate a useful ability to exclude TTH. The right tibial nerve showed the next highest nerve-related AUC, but this result did not remain statistically significant after Holm correction. The exploratory optimal cut-off values were 1.75 kg/cm^2^ and 2.35 kg/cm^2^, respectively. Because these thresholds were derived and evaluated within the same sample, they should be interpreted cautiously and require validation in independent cohorts.

### 4.2. Muscle Tissues Mechanosensitivity

Although women with episodic TTH showed lower PPT values across all evaluated muscle locations, none of the muscle-site differences remained statistically significant after adjustment for age and Holm correction. The right tibialis anterior muscle showed the largest muscular effect size and approached, but did not reach, statistical significance after correction. The left tibialis anterior muscle also showed a comparatively large effect, although its corrected *p*-value was not significant. These results suggest a possible pattern of distal muscle hypersensitivity that requires confirmation in larger samples. Previous evidence has shown changes in trigeminal, extra trigeminal, and distal muscle sensitivity in women with episodic TTH and chronic TTH versus controls [37]. Although this may seem inconsistent with the findings of the present study, when considering the entirety of structures assessed, our results remain aligned with previous research.

A recent systematic review found increased trigeminal and cervical sensitivity as characteristic of episodic TTH, while widespread hypersensitivity is typically observed in chronic TTH, based mainly on studies evaluating muscle tissue rather than nerve tissue [11].

Concerning the ROC curve results, the tibialis anterior showed the highest AUC values among the muscular locations, with the right side slightly outperforming the left. However, neither AUC remained statistically significant after correction for multiple testing. Accordingly, the descriptive group differences observed at muscular locations should not be interpreted as evidence that these measurements can classify individual participants or serve as diagnostic biomarkers. One study analyzing children with episodic TTH which evaluated PPTs in four muscle sites (temporalis, trapezius, interdigital space, and tibialis anterior) did not find PPTs to be reliable discriminators between TTH and controls [18].

Overall, this study compared PPTs across nerve and muscle tissues located in cranio-cervical, upper-limb, and distal lower-limb regions. Although lower PPT values were consistently observed in women with TTH, the overall multivariate group effect was not statistically significant, and only the right greater occipital nerve remained significant in the site-specific analyses after adjustment for age and multiple testing. The ROC analyses similarly showed limited discriminative performance, with the right greater occipital nerve yielding the highest AUC and the only multiplicity-adjusted statistically significant result. Taken together, the findings provide exploratory information regarding the distribution of mechanical pain sensitivity rather than evidence supporting the diagnostic use of multiple site-specific PPT measurements.

### 4.3. Limitations

Although the inclusion of a clinically homogeneous sample of participants with frequent episodic TTH diagnosed according to established criteria represents a strength, several limitations should be acknowledged. First, selection bias cannot be excluded. Participants were recruited from a single university-based center, and enrollment depended on their willingness to participate. Although recruitment was not restricted by sex, only women volunteered, met the eligibility criteria, and were ultimately enrolled. In addition, participants with TTH and headache-free controls were recruited as separate groups rather than from a consecutive, unselected clinical population. This sampling approach may have increased the contrast between groups and potentially overestimated the discriminative performance of the PPT measurements [38]. In addition, residual confounding should also be acknowledged. Body mass index, education, occupation, sleep quality, physical activity, habitual medication and analgesic use were not prospectively collected or standardized and could not be incorporated into the analyses. Menstrual cycle-related variations in experimental mechanical pain sensitivity have been described [39], while habitual caffeine consumption has been associated with differences in PPT values [40]. Diurnal variations in basal pressure pain sensitivity have also been reported [41]. Meteorological conditions on the assessment days were not recorded, and the present study cannot determine whether short-term environmental variation contributed to the variability in PPT measurements. Although recent longitudinal evidence suggests limited and subtype-specific associations between climatic factors and TTH-related healthcare consultations [35], those findings concern healthcare utilization and should not be interpreted as direct evidence of an effect on mechanical pain sensitivity. Therefore, uncontrolled variation in these factors may have contributed to within-group variability and influenced the estimated between-group differences and diagnostic performance. Future studies should record menstrual phase and caffeine consumption and conduct PPT assessments within a predefined time window.

Second, the sample consisted exclusively of young women with frequent episodic TTH. Therefore, the findings cannot be directly generalized to men, older populations, or individuals with chronic TTH. This is particularly relevant because sex-related differences in experimental pain sensitivity have previously been reported [42]. Furthermore, the groups differed significantly in age, with the TTH group being younger than the control group. Although statistical analyses were adjusted for age, results should still be interpreted cautiously and require confirmation in independent samples.

Third, the sample-size calculation was based on detecting between-group differences rather than on estimating AUC values, sensitivity, specificity, or cut-off points with a predefined level of precision. Consequently, the relatively small sample may have limited the precision and stability of the diagnostic estimates. The site-specific cut-off points were also derived and evaluated in the same sample without external validation and should therefore be regarded as preliminary. Although a combination of several anatomically and clinically justified PPT measurements could theoretically provide greater discrimination than an individual site, the present sample was insufficient to develop a stable multivariable classification model. Stepwise modelling was therefore not conducted because of the substantial risk of overfitting and optimistic performance estimates. Future adequately powered studies should evaluate prespecified combinations of clinically justified PPT sites using penalized modelling, internal validation, and subsequent external validation in an independent clinical cohort.

Fourth, although the Holm procedure was applied separately to the 20 age-adjusted between-group comparisons and the 20 AUC hypothesis tests, the number of anatomical locations evaluated relative to the sample size reduced statistical power and increased the uncertainty of the site-specific estimates. Only the right greater occipital nerve site remained statistically significant after correction, and the remaining differences should therefore be interpreted as exploratory patterns requiring confirmation in independent samples. In addition, PPT measurements obtained at locations overlying superficial peripheral nerves cannot selectively isolate neural tissue. The pressure stimulus also involves the skin, subcutaneous tissue, fascia, muscle, vascular structures, and other adjacent tissues. Consequently, these measurements should be interpreted as regional pressure sensitivity at nerve-related anatomical sites rather than as direct evidence of altered nerve-tissue mechanosensitivity or peripheral nerve dysfunction. Similarly, lower PPTs across cranio-cervical and distal locations may be compatible with a pattern of widespread pressure hyperalgesia, but a single psychophysical measure cannot establish central sensitization or determine the underlying neurophysiological mechanism. Alternative explanations, including regional tissue characteristics, measurement variability, contextual or psychological influences, and residual confounding, should also be considered.

Fifth, pericranial tenderness was not systematically recorded, and participants could not be classified as having frequent episodic TTH associated with or without pericranial tenderness. This characteristic may influence mechanical sensitivity, particularly at craniofacial and trigeminocervical locations. Its absence from the available data may therefore have contributed to within-group variability and prevented assessment of whether pericranial tenderness modified the observed PPT differences or within-sample discrimination.

Sixth, the cross-sectional design does not allow temporal or causal relationships to be established between altered mechanical pain sensitivity and TTH. Longitudinal studies are needed to determine whether changes in nerve- and muscle-related PPTs precede headache progression, vary with clinical status, or are responsive to treatment.

Finally, the data were collected in 2018, whereas the present analysis and manuscript were prepared several years later. The eligibility criteria, neurological assessment, anatomical measurement locations, and pressure-algometry procedures were established during the original study period. However, the ROC analyses and derivation of site-specific cut-off values were conceived subsequently as secondary post hoc analyses of the existing dataset and were not specified in a prospectively registered diagnostic-accuracy protocol. This temporal separation increases the importance of transparent reporting and limits the confirmatory interpretation of the ROC findings. Although the original group classifications and measurements were retained and the statistical results were audited against the available output, the exploratory cut-offs should be validated using prospectively collected contemporary data and a prespecified analysis plan.

## 5. Conclusions

Women with frequent episodic TTH showed descriptively lower PPTs across the evaluated cranio-cervical, upper-limb, and distal lower-limb locations than headache-free controls. However, the overall multivariate group effect was not statistically significant after adjustment for age, and only the anatomical site overlying the right greater occipital nerve remained statistically significant after Holm correction. Because laterality was not prespecified and TTH is generally a bilateral disorder, this isolated right-sided finding may reflect sampling variation and should not be interpreted as evidence of a lateralized pathophysiological mechanism.

The principal contribution of this study is the descriptive and age-adjusted comparison of mechanical pain sensitivity across muscular and nerve-related anatomical sites. PPT measurements obtained over superficial nerve courses should be interpreted as regional pressure-sensitivity measures rather than selective assessments of nerve tissue, because the pressure stimulus also involves skin, subcutaneous tissue, fascia, muscle, vascular structures, and other adjacent tissues. Similarly, lower PPTs across cranio-cervical and distal locations may be compatible with a pattern of widespread pressure hyperalgesia, but pressure algometry alone cannot establish central sensitization or determine the neurophysiological mechanism underlying the observed differences.

The ROC analyses should be regarded as secondary and exploratory. The right greater occipital nerve site yielded the highest observed AUC in this sample and was the only location whose AUC remained significantly different from 0.50 after correction for multiple testing. However, no formal pairwise comparisons between AUCs were conducted, and this result does not demonstrate statistically superior performance compared with the other anatomical locations. At the sample-derived cut-off of 1.75 kg/cm^2^, this site showed high specificity but low sensitivity. Although the LR+ was comparatively large, its extremely wide confidence interval indicated substantial imprecision and statistical instability, while the LR− did not support a clinically useful ability to exclude TTH. Consequently, these findings should not be interpreted as evidence of a reliable rule-in or rule-out test.

PPTs should therefore be considered complementary psychophysical measures for characterizing regional and potentially widespread mechanical pain sensitivity rather than stand-alone diagnostic tests. The reported cut-offs and operating characteristics represent apparent performance within a selected derivation sample of young women with established frequent episodic TTH and headache-free controls. Their reproducibility and clinical relevance require confirmation in larger and more representative cohorts that include relevant alternative headache diagnoses. Future adequately powered derivation studies may also evaluate prespecified combinations of anatomically and clinically justified PPT sites using penalized modelling and appropriate internal validation, followed by external validation in independent clinical samples.

## Figures and Tables

**Table 1:** Clinical characteristics observed in subjects with frequent episodic TTH.

	TTH (*n* = 31)
Years of TTH history	2.78 ± 1.78
Days per month with TTH	7.74 ± 3.48
Hours per day with TTH	1.38 ± 0.85
Points of TTH intensity per attack (NPRS)	5.43 ± 1.12
HDI	29.20 ± 9.69
HADS-A score	6.32 ± 1.97
HADS-D score	1.13 ± 1.33

**Table 2:** PPT differences observed between subjects with frequent episodic TTH and asymptomatic controls.

Pressure Pain Thresholds (kg/cm^2^)	TTH (*n* = 31)	Controls (*n* = 32)	Difference (95%CI)	ANCOVA	Holm-Adjusted *p* Value
Left temporalis muscle	2.09 ± 0.85	2.47 ± 0.95	0.54 (0.09; 0.99)	F = 5.746; ηp^2^ = 0.087	0.182
Left C5/C6 zygapophyseal joint	1.74 ± 0.65	2.27 ± 1.52	0.63 (0.02; 1.24)	F = 4.268; ηp^2^ = 0.066	0.228
Left 2nd and 3rd interdigital hand space	2.32 ± 1.08	3.01 ± 1.92	0.99 (0.23; 1.76)	F = 6.697; ηp^2^ = 0.100	0.169
Left tibialis anterior muscle	3.58 ± 1.85	4.74 ± 2.37	1.50 (0.43; 2.57)	F = 7.910; ηp^2^ = 0.116	0.106
Left greater occipital nerve	2.18 ± 0.96	2.65 ± 1.19	0.58 (0.02; 1.14)	F = 4.320; ηp^2^ = 0.067	0.228
Left radial nerve	1.89 ± 0.89	2.31 ± 1.26	0.62 (0.08; 1.16)	F = 5.315; ηp^2^ = 0.081	0.197
Left ulnar nerve	2.50 ± 1.07	3.42 ± 2.30	1.18 (0.26; 2.09)	F = 6.608; ηp^2^ = 0.099	0.169
Left median nerve	1.61 ± 0.55	1.93 ± 1.07	0.47 (0.04; 0.90)	F = 4.788; ηp^2^ = 0.074	0.228
Left tibialis posterior nerve	2.65 ± 1.07	3.26 ± 1.70	0.77 (0.04; 1.50)	F = 4.443; ηp^2^ = 0.069	0.228
Left fibular nerve	2.35 ± 0.97	3.07 ± 1.71	0.98 (0.29; 1.67)	F = 8.083; ηp^2^ = 0.119	0.104
Right temporalis muscle	2.17 ± 0.74	2.51 ± 0.93	0.47 (0.04; 0.89)	F = 4.779; ηp^2^ = 0.074	0.228
Right C5/C6 zygapophyseal joint	1.77 ± 0.62	2.15 ± 0.96	0.50 (0.09; 0.91)	F = 5.897; ηp^2^ = 0.089	0.182
Right 2nd and 3rd interdigital hand space	2.53 ± 1.15	3.23 ± 1.60	0.94 (0.24; 1.63)	F = 7.233; ηp^2^ = 0.108	0.139
Right tibialis anterior muscle	3.62 ± 1.60	4.79 ± 2.24	1.50 (0.52; 2.48)	F = 9.453; ηp^2^ = 0.136	0.057
Right greater occipital nerve	2.24 ± 0.87	2.84 ± 0.95	0.74 (0.28; 1.20)	F = 10.270; ηp^2^ = 0.146	0.043
Right radial nerve	2.05 ± 1.02	2.32 ± 1.09	0.53 (0.04; 1.02)	F = 4.637; ηp^2^ = 0.072	0.228
Right ulnar nerve	2.74 ± 1.28	3.42 ± 1.74	0.96 (0.20; 1.72)	F = 6.399; ηp^2^ = 0.096	0.169
Right median nerve	1.83 ± 0.76	2.12 ± 1.00	0.51 (0.10; 0.93)	F = 6.139; ηp^2^ = 0.093	0.177
Right tibialis posterior nerve	2.59 ± 0.97	3.35 ± 1.54	0.99 (0.35; 1.63)	F = 9.590; ηp^2^ = 0.138	0.056
Right fibular nerve	2.57 ± 1.10	2.95 ± 1.54	0.58 (−0.10; 1.26)	F = 2.909; ηp^2^ = 0.046	0.228

Values are presented in kg/cm^2^. Group values are unadjusted means and standard deviations. Between-group comparisons were conducted using analysis of covariance, with group as the fixed factor and age as the covariate. Adjusted mean differences represent controls minus TTH and were estimated at the sample mean age of 20.90 years; therefore, positive values indicate lower PPTs in the TTH group. The reported 95% confidence intervals correspond to the age-adjusted mean differences and are not adjusted for multiplicity. Partial eta squared (ηp^2^) is reported as the effect-size estimate. *p* values were adjusted across the 20 side-specific anatomical locations using the Holm procedure. Bold indicates statistical significance after correction for multiple testing.

**Table 3:** Discriminant capacity of PPTs at different locations to differentiate patients with frequent episodic TTH from asymptomatic individuals.

Variables	ROC Value	95% CI	Cut-Off Point	Holm-Adjusted *p* Value	Sensitivity	Specificity	Youden Index	Positive LR	Negative LR
Left temporalis muscle	0.640	0.502–0.778	1.85	0.752	0.548 (0.360–0.727)	0.719 (0.533–0.863)	0.267	1.950 (1.029–3.696)	0.628 (0.403–0.980)
Left C5/C6 zygapophyseal joint	0.638	0.501–0.774	1.75	0.752	0.645 (0.454–0.808)	0.562 (0.377–0.736)	0.208	1.475 (0.920–2.363)	0.631 (0.359–1.109)
Left 2nd and 3rd interdigital hand space	0.613	0.472–0.754	1.85	0.882	0.452 (0.273–0.640)	0.844 (0.672–0.947)	0.295	2.890 (1.183–7.065)	0.650 (0.457–0.925)
Left tibialis anterior muscle	0.654	0.520–0.789	3.65	0.450	0.645 (0.454–0.808)	0.594 (0.406–0.763)	0.239	1.588 (0.969–2.602)	0.598 (0.343–1.040)
Left greater occipital nerve	0.621	0.482–0.761	1.55	0.880	0.355 (0.192–0.546)	0.938 (0.792–0.992)	0.292	5.677 (1.368–23.568)	0.688 (0.522–0.907)
Left radial nerve	0.614	0.474–0.754	1.75	0.882	0.613 (0.422–0.782)	0.625 (0.437–0.789)	0.238	1.634 (0.964–2.770)	0.619 (0.369–1.040)
Left ulnar nerve	0.617	0.478–0.757	1.85	0.882	0.355 (0.192–0.546)	0.906 (0.750–0.980)	0.261	3.785 (1.166–12.283)	0.712 (0.536–0.946)
Left median nerve	0.573	0.429–0.718	1.55	0.940	0.581 (0.391–0.755)	0.656 (0.468–0.814)	0.237	1.689 (0.961–2.971)	0.639 (0.394–1.037)
Left tibialis posterior nerve	0.598	0.458–0.738	3.65	0.882	0.806 (0.625–0.925)	0.375 (0.211–0.563)	0.181	1.290 (0.938–1.775)	0.516 (0.221–1.203)
Left fibular nerve	0.629	0.492–0.766	2.15	0.784	0.548 (0.360–0.727)	0.688 (0.500–0.839)	0.236	1.755 (0.958–3.214)	0.657 (0.418–1.033)
Right temporalis muscle	0.639	0.496–0.781	1.85	0.784	0.452 (0.273–0.640)	0.875 (0.710–0.965)	0.327	3.613 (1.335–9.776)	0.627 (0.444–0.885)
Right C5/C6 zygapophyseal joint	0.634	0.496–0.771	1.85	0.784	0.645 (0.454–0.808)	0.562 (0.377–0.736)	0.208	1.475 (0.920–2.363)	0.631 (0.359–1.109)
Right 2nd and 3rd interdigital hand space	0.633	0.496–0.770	2.70	0.784	0.677 (0.486–0.833)	0.531 (0.347–0.709)	0.209	1.445 (0.929–2.248)	0.607 (0.332–1.112)
Right tibialis anterior muscle	0.662	0.527–0.796	3.05	0.342	0.516 (0.331–0.698)	0.812 (0.636–0.928)	0.329	2.753 (1.240–6.112)	0.596 (0.399–0.888)
Right greater occipital nerve	0.706	0.576–0.839	1.75	0.040	0.419 (0.245–0.609)	0.969 (0.838–0.999)	0.388	13.419 (1.866–96.525)	0.599 (0.442–0.814)
Right radial nerve	0.586	0.444–0.728	1.75	0.940	0.516 (0.331–0.698)	0.719 (0.533–0.863)	0.235	1.835 (0.958–3.517)	0.673 (0.441–1.028)
Right ulnar nerve	0.613	0.472–0.754	2.35	0.882	0.419 (0.245–0.609)	0.844 (0.672–0.947)	0.263	2.684 (1.085–6.637)	0.688 (0.493–0.961)
Right median nerve	0.581	0.439–0.722	1.35	0.940	0.323 (0.167–0.514)	0.844 (0.672–0.947)	0.166	2.065 (0.796–5.355)	0.803 (0.604–1.068)
Right tibialis posterior nerve	0.654	0.520–0.789	2.35	0.450	0.484 (0.302–0.669)	0.781 (0.600–0.907)	0.265	2.212 (1.046–4.678)	0.661 (0.449–0.973)
Right fibular nerve	0.564	0.421–0.707	3.00	0.940	0.742 (0.554–0.881)	0.406 (0.237–0.594)	0.148	1.250 (0.877–1.780)	0.635 (0.306–1.317)

Frequent episodic TTH was defined as the positive condition, and PPT values below the reported cut-off were classified as positive. Exact binomial 95% confidence intervals were calculated for sensitivity and specificity. Confidence intervals for positive and negative likelihood ratios were calculated from the corresponding 2 × 2 contingency tables using the log-transformation method. *p* values for the AUC tests were adjusted across the 20 anatomical locations using the Holm procedure. The reported operating characteristics represent apparent performance within the derivation sample.

## Data Availability

The original contributions presented in this study are included in the article. Further inquiries can be directed to the corresponding authors.
